# Spatial and temporal patterns of smoking prevalence in Ontario

**DOI:** 10.1186/s12889-015-1526-7

**Published:** 2015-02-25

**Authors:** Gang Meng, K Stephen Brown, Mary E Thompson

**Affiliations:** PROPEL Center for Population Health Impact, University of Waterloo, 200 University Avenue West, Waterloo, Ontario N2L3G1 Canada; Ontario Tobacco Research Unit, Toronto, Ontario Canada; Department of Statistics and Actuarial Science, University of Waterloo, 200 University Avenue West, Waterloo, Ontario N2L 3G1 Canada

**Keywords:** Spatio-temporal modeling, Smoking prevalence, Ontario, Canadian Community Health Surveys, Cluster analysis, Spatial distribution

## Abstract

**Background:**

Smoking prevalence varies over time and place due to various social, environmental and policy influences. However, its spatio-temporal patterns at small-area level are poorly understood. This paper attempts to describe spatio-temporal patterns of adult (age > 18) and youth (age 12–18) smoking prevalence at the municipality level in Ontario, Canada and identify potential socio-demographic, environmental, and policy factors that may affect the patterns.

**Methods:**

Multilevel temporal and spatio-temporal models were fitted to the Canadian Community Health Surveys (2000–2008) data. In total, approximately 160,000 respondents 12 years of age and over living in Ontario were used for this analysis.

**Results:**

The results indicate that during the time period 2003–2008, age-sex stratified smoking prevalence dropped for both the adult and youth populations in Ontario. The tendency is more obvious for youth than for adults. Smoking restriction at home is a leading factor associated with the decline of adult smoking prevalence, but does not play the same role for youth smoking. Despite the overall reduction, smoking prevalence varies considerably across the province and inequalities among municipalities have increased. Clusters of high and low smoking prevalence are both found within the study region.

**Conclusion:**

The identified spatial and temporal variations help to indicate problems at the local level and suggest future research directions. Identifying these variations helps to strengthen surveillance and monitoring of smoking behaviours and the evaluation of policy and program development at the small-area level.

## Background

Tobacco use persists as the number one cause of preventable disease and death in many parts of the world, including Ontario [1]. In association with increased recognition of the harmful health consequences of smoking and increased legislation and policies against smoking, smoking prevalence has decreased consistently in the United States and Canada in recent years. On the other hand, international evidence shows that, in response to increased marketing restrictions, tobacco companies have increased availability of outlets selling tobacco in socially deprived neighbourhoods [2,3], and promotion of tobacco products in specific areas [4]. In Canada, the production and sale of contraband tobacco products has become widespread [5,6]. All these trends may undermine the effectiveness of tobacco control policies and result in a rebound or a halt to the decline of smoking prevalence. Accurate estimation of smoking prevalence over time and over small areas is important for measuring progress towards anti-smoking objectives, revealing underlying social and environmental determinants, evaluating current anti-smoking campaigns and policies, and planning for specific area-based anti-smoking programs.

Previous studies have identified that smoking behaviours are not only determined by numerous individual-level factors, but are also affected by various social, economic, environmental and policy factors. For example, it is found that supermarkets and convenience stores (the major retailers of tobacco) are more accessible [7] and more concentrated [8] in socially deprived neighbourhoods, and more tobacco advertisements are found in lower socioeconomic communities [9]. Tobacco companies target their advertising to more predominantly minority communities [10,11]. The number of agents displaying no-smoking signs or providing information discouraging smoking may affect the smoking rate in a jurisdiction [12]. Neighbourhood violence [13], socio-economic disadvantage [14,15] and social disorganization [16] may be associated with high smoking prevalence. Urban–rural differences [17] and ethnic spatial segregation [18] may result in significantly different rates of smoking. Tobacco control interventions and policies, such as smoking restrictions in workplaces [19], schools [20], communities and homes [21], restrictions on sales to minors [22], health warnings on tobacco products [23], cigarette price increases [24], and community anti-smoking programs [25], may all lead to smoking behavioural changes. These identified social, environmental and policy-related determinants suggest that smoking prevalence may vary significantly over time and space.

However, unequal changes of small-area patterns of smoking prevalence over time and the extent to which social and environmental determinants may affect the inequalities remain largely under-explored. Only a few studies have attempted to describe the contribution of geography to the total variation of smoking in Canada, and potential explanations of such variation by individual, socioeconomic, demographic characteristics, and family anti-smoking norms [26,27].

The purpose of the paper is to evaluate current adult (age over 18) and youth (age 12 to 18) smoking prevalence and spatio-temporal trends over recent years at the municipality-level in Ontario, Canada, and to identify socio-demographic, environmental, and policy determinants that may affect the patterns. The revealed patterns and potential determinants may not only depict the status quo of smoking behaviours in Ontario, but may also predict the risky areas and point out directions for policy decision making to reduce the prevalence and inequalities of smoking among small areas.

## Methods

### Data

Data on 165,372 respondents from 2000 to 2008 in Ontario, Canada were collected in the Canadian Community Health Surveys (CCHS) (cycles 1.1, 2.1, 3.1, 2007, and 2008). The CCHS is a repeated cross-sectional survey that collects information related to health status, health behaviours (including smoking), community-oriented health determinants and health care utilization for the Canadian population. The first cycle of CCHS started in 2000 and the data were collected for both 2000 and 2001. The second cycle data were collected in 2003 while the third cycle data were collected in 2005. The surveys after 2006 were conducted yearly.

In Ontario, about half of the sample respondents were selected from an area frame and the other half from a list frame of telephone numbers. A stratified two-stage design established for the Canadian Labour Force Survey (LFS) was used for the area frame, while a random sampling process was used given a telephone list in each health region. A full description of the sampling methods is available online at Statistics Canada’s website [28]. Based on this sampling design, although samples are not uniformly distributed among small areal units (smaller than health regions), almost all the census sub-divisions (CSDs) contain enough respondents for the estimation of smoking prevalence at this level. Since CSDs are deemed to be equivalent to municipalities of Canada, the data provide an important opportunity to examine the spatial and temporal patterns and determinants of smoking prevalence among municipalities.

Respondents’ ages in the collected CCHS data range from 12 to 102. Smokers were defined as individuals who had smoked more than 100 cigarettes in their lifetimes, and smoked at least once in the previous 30 days. In addition to smoking status, the data contain age, gender, socio-demographic factors, psycho-social factors, policy related variables, geographical locations, and geographical identifiers (postal codes). Variables used in the current analysis were described in Table 1. Since this is a secondary analysis of Statistics Canada data, no ethics clearance is required by the Office of Research Ethics at the University of Waterloo. All security procedures required by Statistics Canada to access and use the data for analysis were followed.

**Table 1:** **Variable description**

**Variable**	**Description**
**Response variable:**	
Smoking status	Defined as 1- individuals who had smoked more than 100 cigarettes in their lifetimes, and smoked at least once in the previous 30 days; and 0 – otherwise.
Successful cessation	Defined as 1 - smokers who successfully quit in the last year and more than a year ago; and 0 – otherwise.
**Exposure variables:**	
*AGE*	Age at the time of survey
*SEX*	1 – female; 2 – male
Marital status (*MS*)	1 – married or common law; 0 – otherwise
Family income (*INCOME*)	Standardized household income with 0 mean and 1 variance.
Unemployment (*UNEMPLOY*)	1 - Full-time or patricianly employed; 0 – otherwise.
Low education (*LOWEDU*)	1- High school or lower; 0- otherwise.
Perceived life stress (*PLS*)	Perceived life stress: 1- Not at all stressful, 2- not very stressful, 3 - a bit stressful, 4 - quite a bit stressful, and 5 - extremely stressful.
Sense of belonging to communities (*SBC*)	1- very strong , 2- somewhat strong, 3- somewhat weak, and 4 - very weak.
Complete workplace smoking restrictions (*SMKRWC*)	1- completely restricted smoking restrictions at place of workplace; 0- otherwise.
Partial work place smoking restrictions (*SMKRWP*)	1- allowed in designated areas or restricted only in certain places; 0 – otherwise.
Home smoking restrictions (*HOME_RESTRIC*)	Restrictions against smoking cigarettes in home: 1 - Yes 1, 0 – no.
Geographic locations (*GEO*)	Defined as 1- Greater Toronto Area (GTA); 2- any other urban areas; and 3- rural area.
*YEAR*	Survey year: 0 – 2000; 1 – 2001; … ; 8 – 2008;

### Temporal and spatio-temporal analyses

To analyze the seemingly downward overall time trend of smoking prevalence in Ontario and potential affecting factors, multi-level temporal models were constructed and fitted using the SAS v9.2 GLIMMIX procedure. Since adults and youth smoking behaviours may be affected by different risk factors, adult (age 19 and over, including 147,118 respondents) and youth (age 12 – 18, including 18,254 respondents) populations were analyzed separately. Assuming that the time trend of smoking prevalence is not linear over the years, the full temporal models are defined as follows.

For adult *i* in census subdivision *j*:

1 $$ \begin{array}{l} Adult\  smoking\  status \sim binary\ \left({p}_{ij}\right)\\ {} Level\ 1\ \left( person\  level\right):\  logit\left({p}_{ij}\right) = {\beta}_{0j} + {\beta}_1AG{E}_{ij} + {\beta}_2SE{X}_{ij} + {\beta}_3M{S}_{ij} + {\beta}_4 INCOM{E}_{ij} + {\beta}_5 UNEMPLO{Y}_{ij} + {\beta}_6\\ {}\kern1em  LOWED{U}_{ij} + {\beta}_7 PL{S}_{ij} + {\beta}_8SB{C}_{ij} + {\beta}_9 SMKRW{C}_{ij} + {\beta}_{10} SMKRW{P}_{ij} + {\beta}_{11} HOME\_ RESTRI{C}_{ij} + {\beta}_{12}GEO+\\ {}\kern1em {\beta}_{13}YEA{R}_{ij} + {\beta}_{14}YEA{R_{ij}}^2 + {\beta}_{15}YEA{R}_{ij}* HOME\_ RESTRI{C}_{ij}+{\beta}_{16}YEA{R_{ij}}^2* HOME\_ RESTRI{C}_{ij}\\ {} Level\ 2\ \left( Census\  subdivision\  level\right):\kern0.5em {\beta}_{0j} = {\gamma}_0 + {v}_{0j}\end{array} $$

For youth *i* in census subdivision *j*:

2 $$ \begin{array}{l} Youth\  smoking\  status \sim binary\ \left({p}_{ij}\right)\\ {} Level\ 1\ \left( person\  level\right):\  logit\left({p}_{ij}\right) = {\beta}_{0j} + {\beta}_1AG{E}_{ij} + {\beta}_2SE{X}_{ij} + {\beta}_3 INCOM{E}_{ij}+{\beta}_4 PL{S}_{ij}+{\beta}_5SB{C}_{ij}+{\beta}_6 HOME\_ RESTRI C\\ {}{}_{ij} + {\beta}_7GEO + {\beta}_8YEA{R}_{ij} + {\beta}_9YEA{R_{ij}}^2 + {\beta}_{10}YEA{R}_{ij}* HOME\_ RESTRI{C}_{ij}+{\beta}_{11}YEA{R_{ij}}^2* HOME\_ RESTRI{C}_{ij}\\ {} Level\ 2\ \left( Census\  subdivision\  level\right):\kern0.5em {\beta}_{0j} = {\gamma}_0 + {v}_{0j}\end{array} $$

where smoking status has a binary distribution. The log odds of smoking probabilities are regressed to year, and year squared. For adults, the model at level 1 (individual level) also includes age, sex, marital status (*MS*), family income (*INCOME*), unemployment (*UNEMPLOY*), low education (*LOWEDU*), perceived life stress *(PLS*), sense of belonging to communities (*SBC*), complete and partial work place smoking restrictions (*SMKRWC* and *SMKRWP*), home smoking restrictions (*HOME_RESTRIC),* and geographic locations (*GEO*). The *GEO* variable is included to control for any variations of smoking prevalence between large urban (the Greater Toronto Area), other urban and rural areas. For youth, the model includes age, sex, family income, PLS, SBC, home smoking restriction (*HOME_RESTRIC*), and *GEO*. Assuming that smoking prevalence is different among municipalities, a random intercept was constructed at the census subdivision level with a fixed average effect *γ*_*0*_, and a random effect *v*_*0j*_, which has a normal distribution with a mean of 0.

The time trend was tested by incrementally adding explanatory variables in the above models. The overall time trend was first tested by adding in only the time variables and controlling for age and sex (Model 1). The socio-demographic, socio-economic (SES), psycho-social, and workplace smoking restriction variables were then added to the model to test whether or not these variables may have potential impacts on the time trend (Model 2). The variable of home smoking restrictions was further added (Model 3), followed by adding in the interaction terms of time and home smoking restriction (Model 4) to test the potential impact of home smoking restriction on the time trends. Since only smokers were asked the question on home smoking restrictions in the 2000 and 2001 surveys and all respondents were asked the same question in 2003–2008 surveys, the above models were fitted using the 2003–2008 data only, which include 112,848 adult and 13,863 youth respondents.

To test how spatial dependencies are modeled and whether or not there are remaining spatial autocorrelations, spatial dependencies at the area level were also calculated using the global Moran’s I [29] on the CSD-level residual, *v*_*0j*_, after Equations (1) and (2) were fitted.

Previous research suggests that the extent of home smoking restrictions is one of the most powerful determinants of cessation [21] and may therefore be an important predictor for smoking reduction. To test the association between smoking restriction and adult smoking cessation, a model similar to that of Equation (1) was also constructed with the variable of successful cessation as the outcome and year variables removed.

Based on the results of the above analysis, the distributions of smoking prevalence among municipalities and the changes of these patterns over time were further constructed and tested using multi-level spatial temporal modeling (WinBUGS 1.4.3) [30]. The models for adult and youth were constructed as follows.

*ADULT:*

$$ Smoking\  status \sim binary\ \left({p}_{ij}\right) $$

Level 1 (PERSON LEVEL):

$$ \begin{array}{l} logit\left({p}_{ij}\right) = {\beta}_{0j} + {\beta}_1AG{E}_{ij} + {\beta}_2SE{X}_{ij} + {\beta}_3M{S}_{ij} + {\beta}_4 INCOM{E}_{ij} + {\beta}_5 UNEMPLOYMEN{T}_{ij} + {\beta}_6 LOWED{U}_{ij} + {\beta}_7 PL{S}_{ij}\\ {} + {\beta}_8SB{C}_{ij} + {\beta}_9 SMKRW{C}_{ij} + {\beta}_{10} SMKRW{P}_{ij} + {\beta}_{11j}YEA{R}_{ij} + {\beta}_{12j} HOME\_ RESTRI{C}_{ij}\end{array} $$

Level 2 (CSD LEVEL):

3 $$ \begin{array}{l}{\beta}_{0j} = {\gamma}_0 + {v}_{0j} + {u}_{0j}\\ {}{\beta}_{11j} = {\gamma}_1 + {v}_{1j} + {u}_{1j}\\ {}{\beta}_{12j} = {\gamma}_2 + {v}_{2j} + {u}_{2j}\end{array} $$

*YOUTH:*

$$ Smoking\  status \sim binary\ \left({p}_{ij}\right) $$

Level 1 (PERSON LEVEL):

$$ logit\left({p}_{ij}\right) = {\beta}_{0j} + {\beta}_1AG{E}_{ij} + {\beta}_2SE{X}_{ij} + {\beta}_3 INCOM{E}_{ij} + {\beta}_4 PL{S}_{ij} + {\beta}_5SB{C}_{ij} + {\beta}_6 HOME\_ RESTRI{C}_{ij} + {\beta}_{7j}YEA{R}_{ij} $$

Level 2 (CSD LEVEL):

4 $$ \begin{array}{l}{\beta}_{0j} = {\gamma}_0 + {v}_{0j} + {u}_{0j}\\ {}{\beta}_{7j} = {\gamma}_1 + {v}_{1j} + {u}_{1j}\end{array} $$

The models at level 1 are similar to the corresponding temporal models in Equations (1) and (2). Since the time trend after controlling for identified variables was almost linear (see the Results section), only a single *YEAR* variable (rather than *YEAR* and *YEAR*^*2*^) is included in Equations (3) and (4) for simplicity. The GEO variable is taken out since the effects of geographical locations have already been borne by *u*_*0j*_, *u*_*1j*_ and *u*_*2j*_. At the CSD level, based on the results of the above temporal models, it is assumed that smoking prevalence, the time influence, and smoking restrictions at home may vary among municipalities for adults, and smoking prevalence and the time influence may vary among municipalities for youth. The fixed average effects *γ*_*0*_, *γ*_*1*_, and *γ*_*2*_, the uncorrelated random effects *v*_*0j*_, *v*_*1j*_ and *v*_*2j*_, and the spatially correlated random effects *u*_*0j*_, *u*_*1j*_ and *u*_*2j*_ were used for smoking prevalence, the time influence and smoking restriction at home respectively to analyze the municipal-level variations. Given the generally large sizes of municipalities, spatial dependencies likely only exist among adjacent municipalities. Therefore, an intrinsic conditional autoregression (CAR) model with a contiguity neighbourhood structure (assuming only adjacent neighbourhoods are spatially auto-correlated) was used for *u*_*0j*_, *u*_*1j*_ and *u*_*2j*_ to model the spatial dependencies at the municipal level. After these models were fitted, the spatial variation of smoking prevalence, time influence, and smoking restriction at home can be described using *v*_*0j*_ *+ u*_*0j*_, *v*_*1j*_ *+ u*_*1j*_, and *v*_*2j*_ *+ u*_*2j*_ respectively. Since WinBUGS models allow missing data to be treated as stochastic nodes (values to be estimated), all the data obtained from 2000 to 2008 were used to fit the models. The posterior mean values and random effects were used for estimating the spatio-temporal impacts of smoking prevalence.

It can be seen that the spatial and temporal interactions were explicitly measured by the spatially dependent coefficient of the *YEAR* variable, namely *β*_*11j*_ for adult and *β*_*7j*_ for youth. This coefficient allows spatially unequal changes of smoking prevalence over time to be mapped and dramatic changes to be identified.

Since CCHS is a repeated cross-sectional survey, survey weights were also adjusted for the proposed analysis that pools together data from different cycles. The adjusted weight is constructed as follows:

5 $$ W=WTS\_M* sample\_ size/ sum\_ of\_ sample\_ size s $$

where WTS_M is the CCHS survey weight, sample_size is the sample size of current cycle, and sum_of_sample_sizes is the sum of sample sizes from all cycles being used for the analysis. This adjustment allows samples from different cycles to be comparable. The adjusted weights were applied to the temporal models (Equations 1 and 2) so that the estimates are representative of the population in the study area. Given the inability of the Bayesian models in WinBUGS to incorporate weights, the weights were not applied to the spatio-temporal models for Equations (3) and (4).

## Results

### Temporal and spatio-temporal patterns of adult smoking prevalence

Table 2 shows that the weight adjusted smoking prevalence have been dropped from 26.2% in 2000 to 21.3% in 2008. To examine potential determinants of smoking prevalence and the downward trends, models described in Equations (1) and (2) were fitted and the results were presented in Table 3. The Moran’s I test of global spatial autocorrelation on CSD-level residuals shows that the spatial autocorrelations for four adult model residuals are small, but statistically significant. This may not affect model fitting, but indicate potential existence of local clusters that need to be further examined.

**Table 2:** **Prevalence of smoking and smoking restriction by years, Ontario (2000–2008, CCHS)**

	**Year**						
	**2000**	**2001**	**2003**	**2005**	**2007**	**2008**	**Overall**
**Smoking prevalence (weighted raw percentages):**							
Adult (19 and over)	26.2%	25.7%	23.5%	22.2%	22.3%	21.3%	23.2%
Youth (12–18)	13.8%	13.9%	10.8%	8.8%	7.3%	7.2%	10.0%
Total	24.7%	24.4%	22.1%	20.7%	20.7%	19.7%	21.8%
**Smoking restriction (weighted raw percentages):**							
Home smoking restrictions: *HOME_RESTRIC* = 1	-	-	69.6%	75.3%	78.1%	78.5%	73.1%
Complete smoking restriction at workplace: *SMKRWC* = 1	-	-	46.5%	44.0%	47.2%	45.6%	45.6%
Partial smoking restriction at workplace: *SMKRWP* = 1	-	-	16.7%	14.6%	13.4%	13.0%	14.8%

Note: workplace restrictions were calculated based on self-reported employed respondents

**Table 3:** **Test results on factors affecting smoking trend (2003–2008)**

**Effect**	**Model 1: controlling for age and sex**		**Model 2: further adding in socio-demographic, socio-economic, psycho-social variables and smoking restrictions at work place**		**Model 3: further adding in smoking restriction at home**		**Model 4: further adding in interactions of smoking restriction at home and time**	
	**Adult (19 +)**	**Youth (12–18)**	**Adult (19 +)**	**Youth (12–18)**	**Adult (19 +)**	**Youth (12–18)**	**Adult (19 +)**	**Youth (12–18)**
Intercept	−0.12**	−10.76***	−0.89***	−9.96***	−0.22***	−9.78***	−0.26***	−10.04***
AGE	−0.02***	0.52***	−0.02***	0.38***	−0.03***	0.38***	−0.03***	0.38***
MALE	0.37***	0.03**	0.36***	0.12***	0.34***	0.11***	0.34***	0.12***
YEAR	−0.04***	−0.17***	−0.02***	−0.11***	0.02***	−0.11***	0.06***	−0.09***
YEAR2	0.004***	0.01***	0.002***	0.004**	0.002***	0.01***	−0.01***	0.02***
INCOME			−0.10***	−0.08***	−0.08***	−0.08***	−0.08***	−0.07***
SBC			0.09***	0.27***	0.06***	0.26***	0.06***	0.26***
PLS			0.16***	0.32***	0.16***	0.32***	0.16***	0.33***
Other urban (vs. rural)			0.08***	0.38***	0.09***	0.38***	0.09***	0.37***
GTA (vs. rural)			−0.37	0.07	−0.31	0.09	−0.31	0.06
LOWEDU			0.67***		0.62***		0.62***	
UNEMPLOY			0.66***		0.61***		0.61***	
SMKRWC			−0.02***		0.01*		0.01*	
SMKRWP			0.52***		0.5***		0.5***	
MS			−0.31***		−0.22***		−0.22***	
HOME_RESTRIC					−0.91***	−0.24***	−0.85***	0.1***
YEAR* HOME_RESTRIC							−0.07***	−0.06**
YEAR2* HOME_RESTRIC							0.01***	−0.02***
Moran’s I test of global spatial autocorrelation on CSD-level residuals	I = 0.129**	I = −0.06	I = 0.125**	I = −0.06	I = 0.109**	I = −0.06	I = 0.109**	I = −0.06

*Significant at p<.05; **Significant at p<.01; ***Significant at p<.0001.

**Figure 1:**
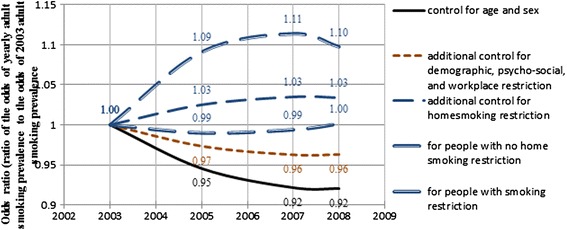
**Temporal trends for adult smoking prevalence (2003–2008).**

**Figure 2:**
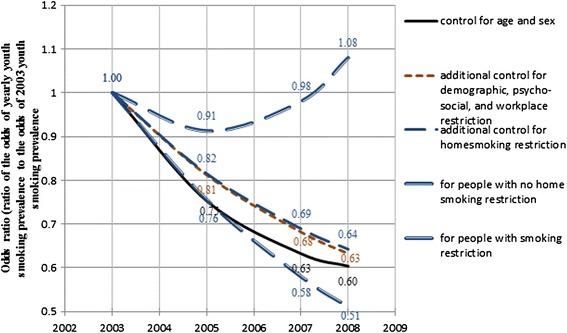
**Temporal trends for youth smoking prevalence (2003–2008).**

Using the odds of smoking prevalence in 2003 as the baseline and the fixed coefficient estimates for *YEAR* and *YEAR*^*2*^, Figures 1 and 2 present the temporal trends for adult and youth smoking prevalence between 2003 and 2008. In these figures, five estimated time trends are presented. The predicted percentage changes presented hereafter are calculated by balanced prevalence; i.e. the predicted smoking prevalence averaged across all levels of the corresponding controlling variables.

Figure 1 shows that the rate of decline of adult smoking prevalence has slowed down over the years. From 2003 to 2008, the odds ratio goes down to 0.92, representing a reduction of the balanced adult smoking prevalence by 1.54%. The downward trend is somewhat reduced to 0.7% (odds ratio goes down to 0.96) after controlling for variables of SES, psycho-social variables and workplace restrictions, indicating some potential impact of these variables on the reduction of smoking prevalence. The downward trend is reversed to have a 0.6% increase (odds ratio goes up to 1.03) after further controlling for the home smoking restriction variable. This indicates that home smoking restrictions may account for about 1.3% of the adult smoking reductions over the five years between 2003 and 2008.

In model 4 of Equation (1), the interaction terms between the two time variables (*YEAR* and *YEAR*^*2*^) and smoking restriction at home are statistically significant, indicating some potential change of the impacts of smoking restriction at home on adult smoking prevalence over the years. However, compared to the main effect (−0.8458), the interactions are relatively small. The two interaction terms (YEAR*HOME_RESTRIC and *YEAR*^2^* HOME_RESTRIC) averaged out and made the changing impacts over the years relatively even. For adults with smoking restrictions at home, the odds ratio is nearly the same between 2003 and 2008, indicating that there is no obvious change of smoking prevalence over these years for adults with smoking restrictions at home. For adults without smoking restrictions at home, the odds ratio goes up by 0.1 from 2003 to 2008, representing a 2.1% increase of smoking prevalence. Therefore, since the smoking prevalence did not change for adults in an environment with home smoking restrictions, but increased in an environment without home smoking restrictions, the overall downward trend of adult smoking prevalence must be associated with the increase in smoking restricted homes over these years. The data (Table 2) also show that home smoking restrictions increased from 69.6% in 2003 to 78.5% in 2008.

Given the above results that home smoking restriction may explain the downward trend of adult smoking, a further test on the association between smoking restriction and cessation was conducted. The result shows that partial workplace smoking restriction (0.155, P < 0.001), complete workplace smoking restriction (0.036, P < 0.0001) and home smoking restrictions (0.82, P < 0.0001) are all positively associated with adult successful cessations after controlling for age, sex, SES, marital status, psycho-social factors, and geography. While the result confirms the associations between smoking restrictions and successful cessations, the downward trend of smoking prevalence is only associated with smoking restrictions at home, possibly due to the increased prevalence of smoking restriction at home, but not at the workplace, in the period under study. Table 2 does confirm that while home smoking restrictions increased, there was no obvious change for smoking restrictions at workplaces over the years 2003–2008.

**Figure 3:**
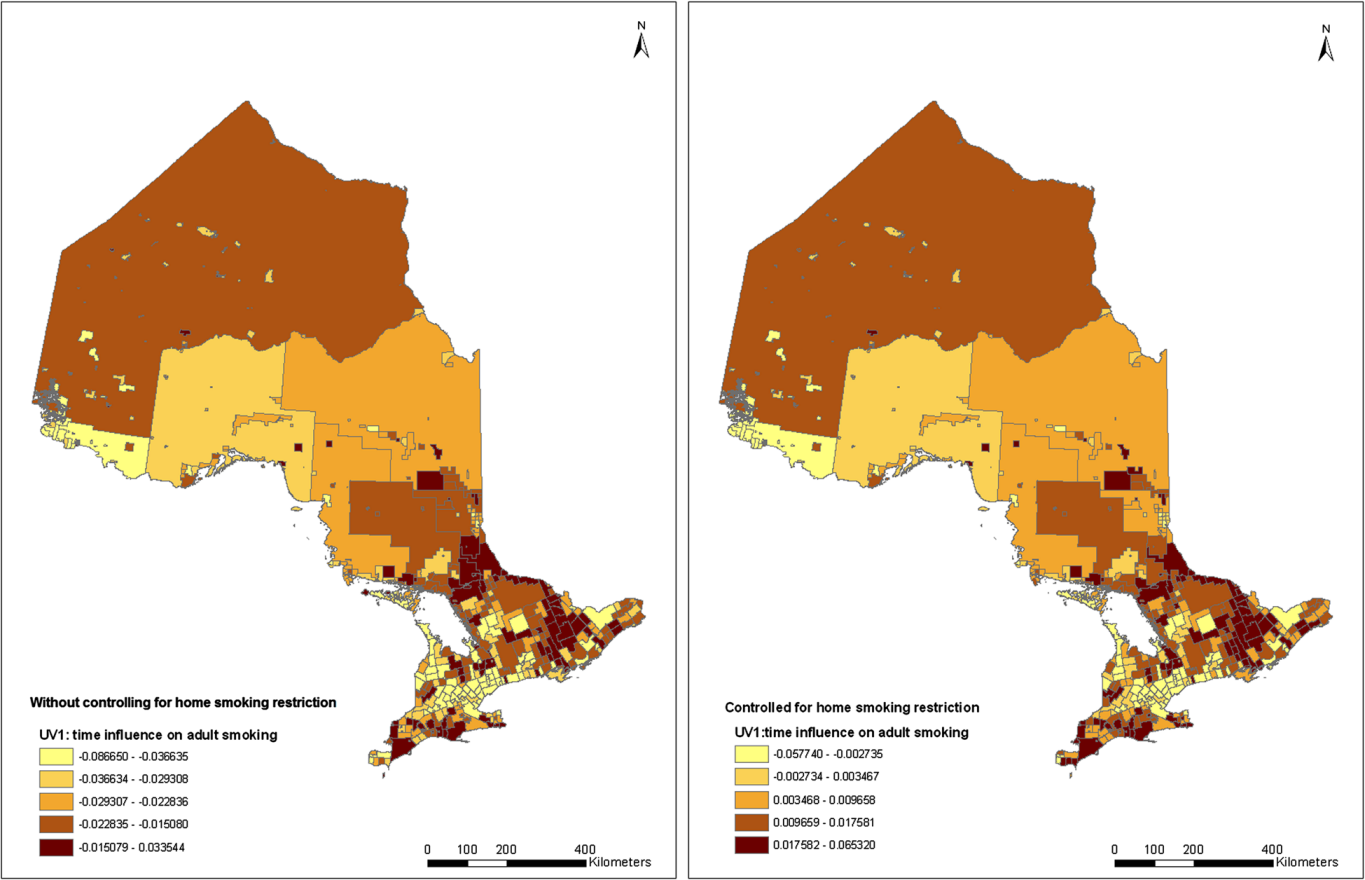
**Quintile distribution of CSD-level random time impacts on adult smoking without and with controlling for home smoking restrictions (2000–2008).**

To investigate how adult smoking prevalence and the impact of smoking restriction at home change over time and space, the spatio-temporal pattern of adult smoking was estimated using Equation (3). The spatial distribution of the estimated random effects for the *YEAR* parameter, *v*_*1j*_ *+ u*_*1j*_, without and with adding in the home smoking restriction variable, are demonstrated in the two maps in Figure 3. The spatial patterns in the first map in Figure 3 show that smoking rate changes differently from municipality to municipality. After controlling for known factors, adult smoking reduction is found largely around large metropolitan areas, including the GTA and Ottawa, and the northwestern part of Ontario. The northwestern area with a relatively light color shown in the map is Rainy River and several other surrounding CSDs, which contain 1105 adult respondents in the data. A potential “route” of increased smoking rate can be observed starting from the east corner of Ontario (Cornwall city) and extending to Northern Ontario (around the city of Greater Sudbury) along the Ottawa valley. A few other areas of increased smoking rates can also be observed on south-western Ontario along Lake Erie.

Comparing the two maps in Figure 3, although the changes of smoking rates are different among municipalities, the spatial patterns are almost the same for the two maps, suggesting that there is no particular effect of home smoking restriction clustering in certain areas. A relatively higher value in each category is seen in the latter map compared to the former. This is consistent with the result in Table 3 that smoking restrictions have a potential impact that contributes to the changes in adult smoking prevalence over the years. Thus, smoking restrictions at home may have increased evenly among municipalities over the years.

**Figure 4:**
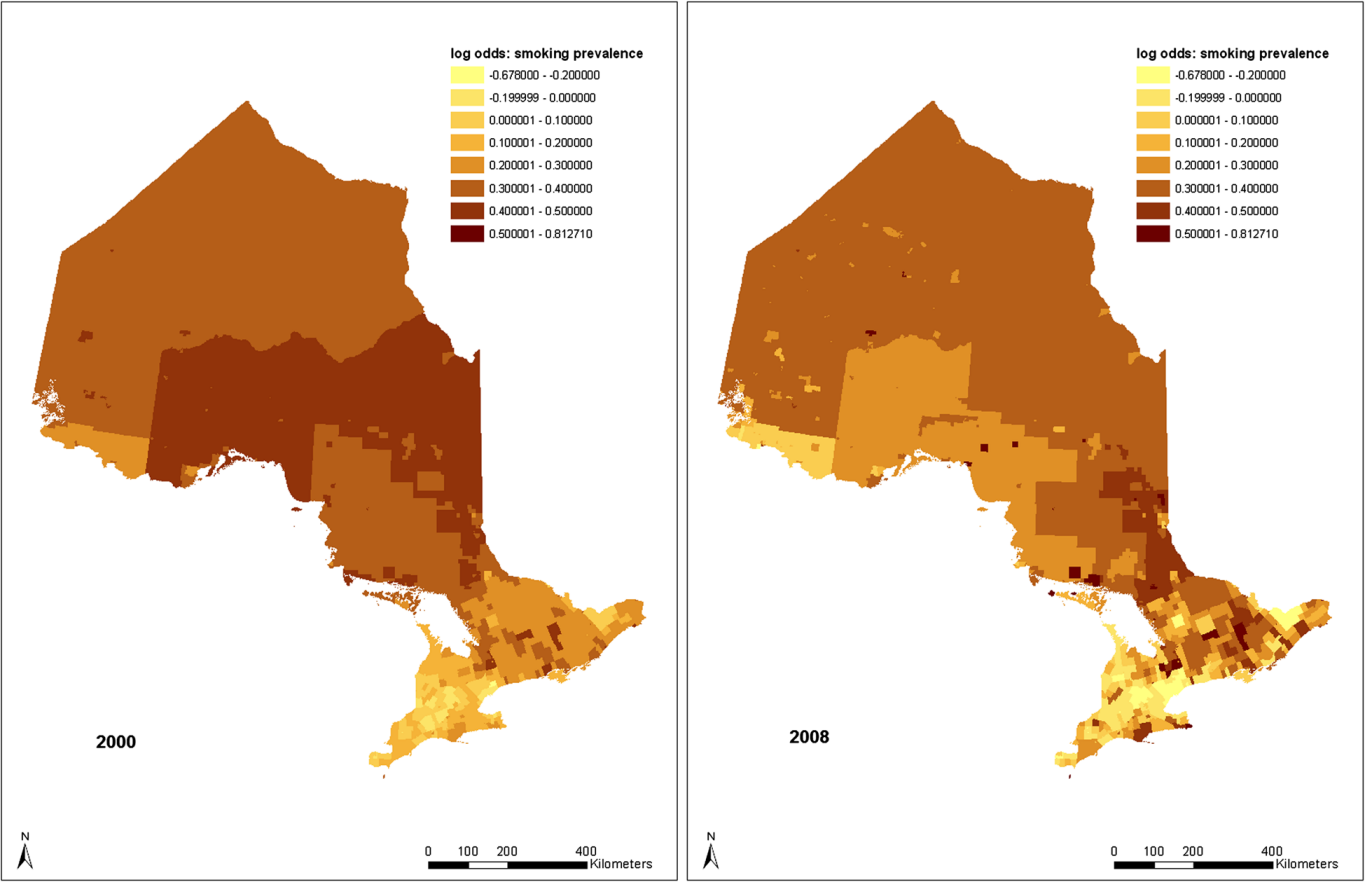
**CSD level predicted distributions of adult smoking prevalence in 2000 and 2008 without controlling for home smoking restriction.**

Figure 4 shows how these time changes affect the pattern of adult smoking prevalence from 2000 to 2008. The overall trend shows that smoking prevalence gradually increases as location moves to the north. It can be seen that the lowest smoking rates are still around the GTA and Ottawa. The Rainy River area still shows a relatively low smoking rate. As has been shown in the time influence map (Figure 3), the highest smoking prevalence has moved toward the Ottawa Valley area by 2008. However, the pattern in 2008 is not as clear as it is in 2000. Smoking inequalities among CSDs increased although overall smoking rates decreased. The random effect (*v*_*0j*_ *+ u*_*0j*_) ranges from −0.678 to 0.813, representing a variation of the balanced smoking prevalence (predicted smoking prevalence averaged across all levels of the explanatory variables) from 15.4% to 44.7%.

**Figure 5:**
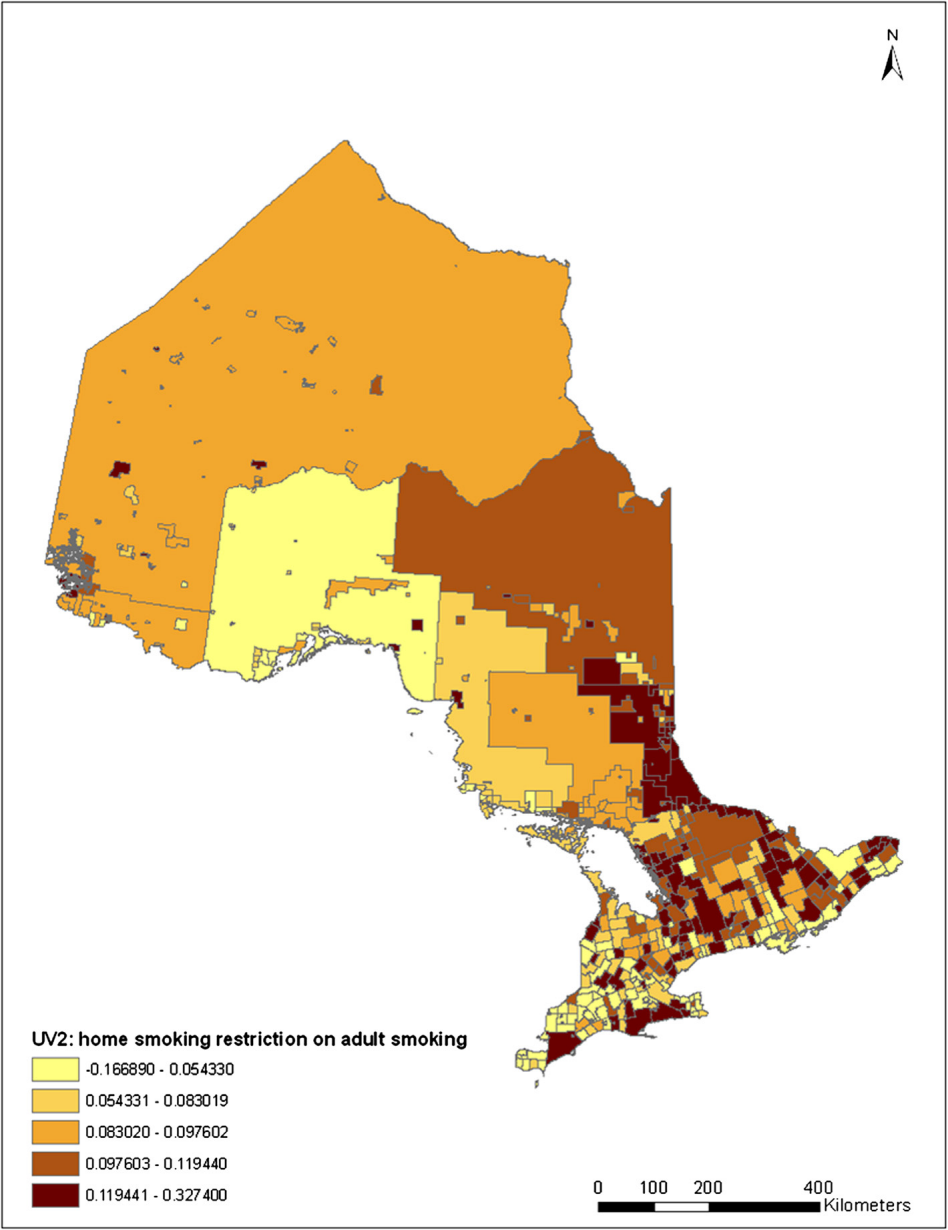
**Quintile distribution of impact of smoking restriction at home on adult smoking (2000–2008).**

Although home smoking restriction may increase evenly among municipalities and its impact on smoking rates may not change over the years, the impact may not be the same from municipality to municipality. Figure 5 shows the distribution of the random effect of home smoking restriction on adult smoking prevalence among municipalities. It can be seen in this figure that the pattern is somewhat consistent with the pattern of adult smoking prevalence in 2008 (Figure 4) and the pattern of time impacts (Figure 3). While Figure 3 indicates that the presence of home smoking restrictions does not affect the time influence on adult smoking, the spatial distribution of home smoking restrictions is related to the distribution of smoking prevalence over municipalities. The similarity of Figures 3 and 5 may indicate that there may be factors that affect both smoking rates and smoking restrictions at home.

### Temporal and spatio-temporal patterns of youth smoking prevalence

For youth smoking, Table 2 shows that the weight adjusted smoking prevalence has dropped from 13.8% in 2000 to 7.2% in 2008. Models similar to those for adults were fitted and the results were presented in Table 3. The global Moran’s I test shows that the spatial autocorrelations for the four youth model residuals are not statistically significant, indicating a good fit of the models accounting for spatial dependencies.

The curves in Figure 2 show a similar pattern of decrease for youth smoking prevalence. The balanced prevalence goes down by 2% (odds ratio goes down to 0.6) from 2003 to 2008. However, the downward trend does not actually change after adding in household income, sense of belonging to local community, perceived life stress and home smoking restrictions (odds ratio = 0.63, representing a balanced prevalence decrease of 1.9%). The two interaction terms, YEAR*HOME_RESTRIC and *YEAR*^2^*HOME_RESTRIC, do show that there is a potential increased effect of home smoking restriction on youth smoking prevalence over the years. For youth with home smoking restrictions, prevalence goes down by 2.9% (odds ratio = 0.51) from 2003 to 2008. For youth without home smoking restrictions, prevalence goes down first and goes back up again to a final increase of 0.4% (odds ratio = 1.08) in 2008. Although home smoking restriction does not explain the downward trend of youth smoking, the potential restrictive impact on youth smoking of a home environment with smoking restriction does increase over these years.

**Figure 6:**
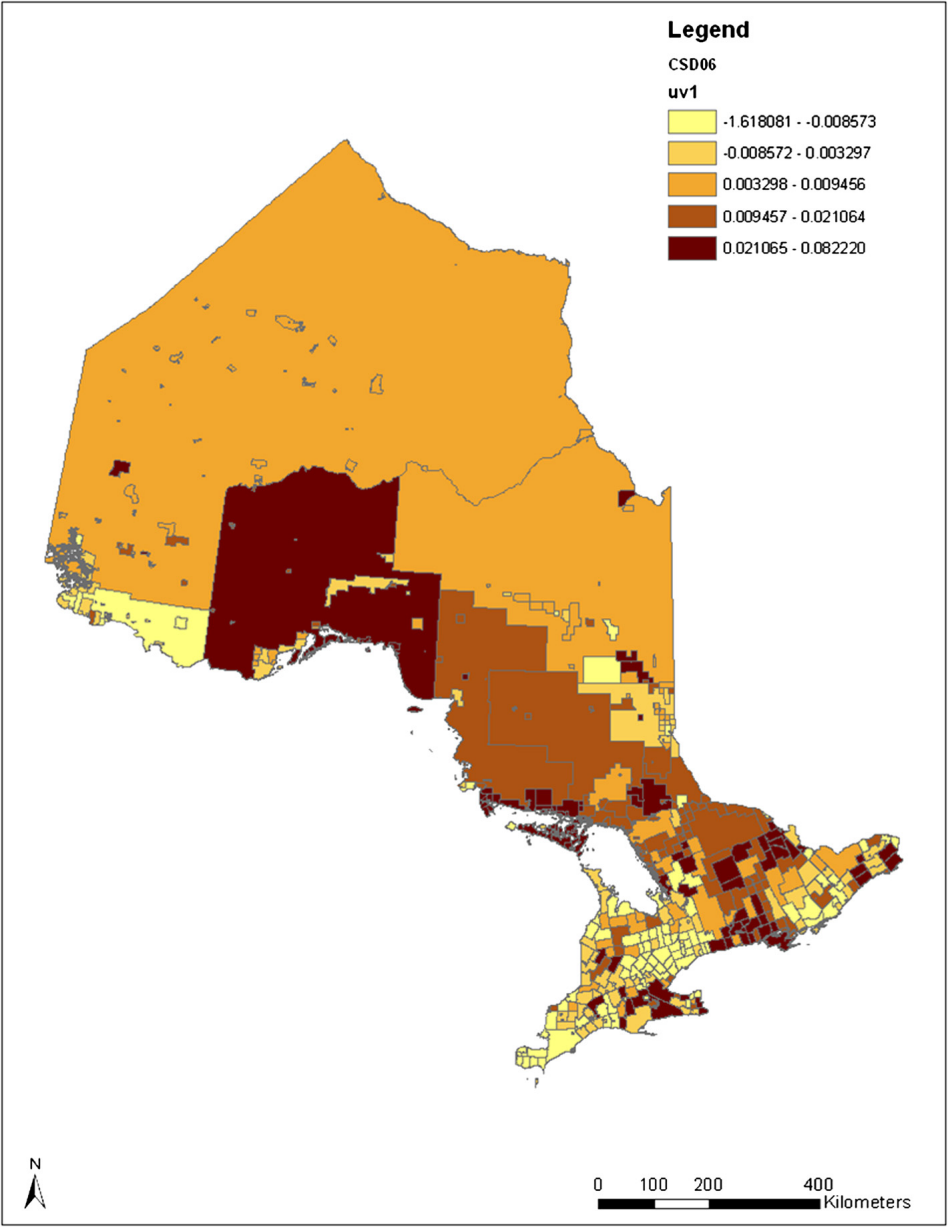
**Quintile distribution of CSD-level random time impact on youth smoking (2000–2008).**

The spatial distribution of the random time influence, *v*_*1j*_ *+ u*_*1j*_, estimated using Equation (4), is mapped in Figure 6. The map shows somewhat different patterns than adult time influence (Figure 3). It can be seen that the highest youth smoking reduction over the years is around the GTA, Essex County, the City of Kingston, the City of Timmins, and the Town of Rainy River. Several areas have the highest youth smoking increases, including areas around Brantford (where reserves marketing cigarettes are located), the counties of Hastings and Prince Edward, and a few other areas in Northern Ontario.

**Figure 7:**
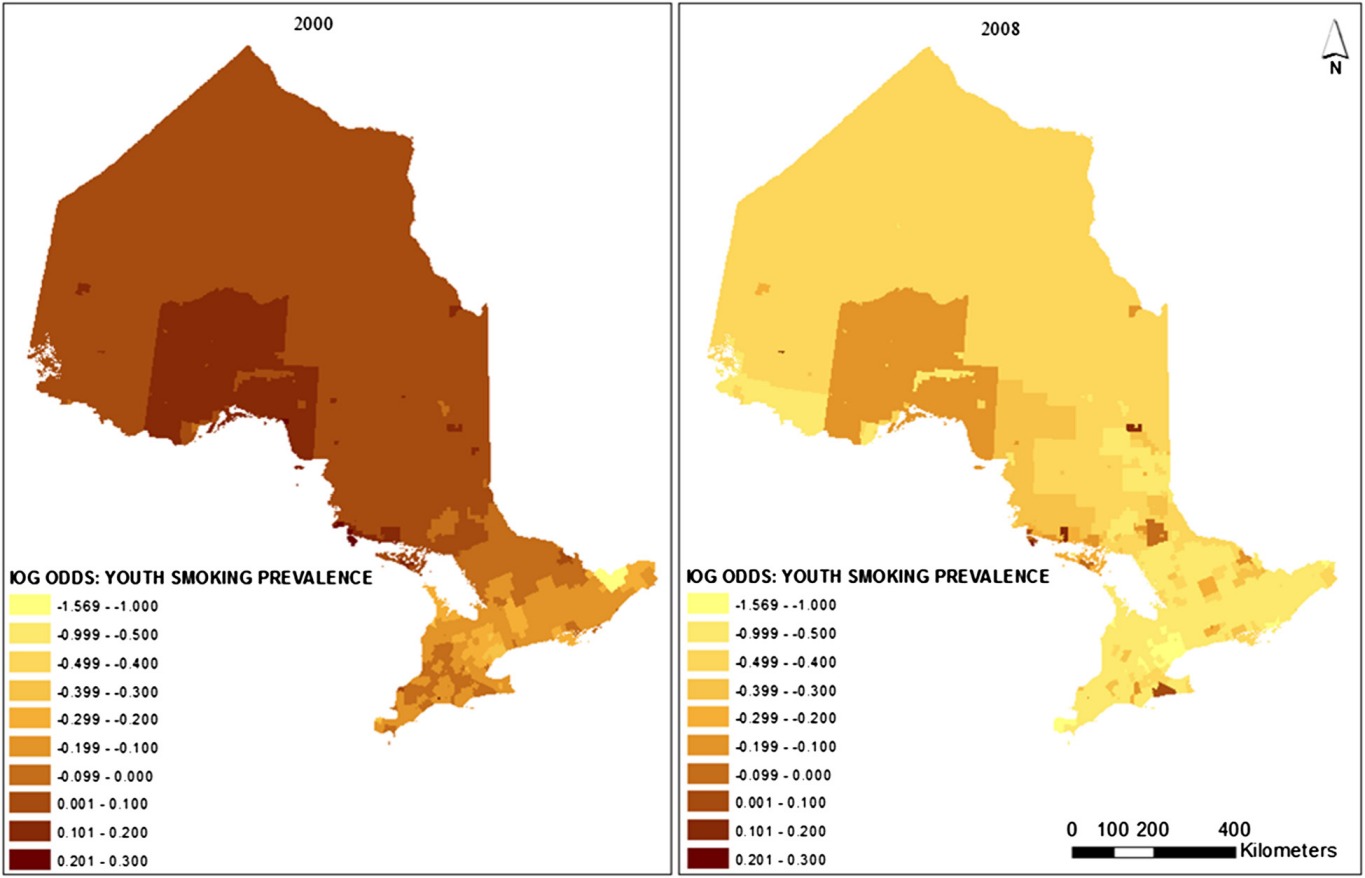
**CSD level predicted distributions of youth smoking prevalence in 2000 and 2008.**

Figure 7 shows the CSD-level changes of youth smoking prevalence from 2000 to 2008. It can be seen that while the overall smoking prevalence is reduced, the pattern does not have significant changes. The overall pattern shows that youth smoking rates are lower in the south than in the north. In 2008, higher smoking rates can be found in the Thunder Bay and Algoma districts, around the Brantford area, and somewhat along the Ottawa Valley. The range of the log odds differences in smoking rates is from −1.57 to 0.3, representing a balanced percentage change from 2.7% to 15.4%. Unlike adult smoking prevalence, youth smoking shows a somewhat reduced inequality over the years. This may indicate potential success of provincial level youth smoking intervention programs or policies.

## Discussion and conclusions

The case study analysis shows that both adult and youth smoking prevalence have been declining in Ontario in the recent decade (Table 2). In addition to the raw prevalence, comparing to the solid black lines (Model 1) in Figures 1 and 2, youth prevalence shows a faster reduction than adult prevalence. This trend may indicate the success of youth smoking prevention strategies, programs or policies in Ontario in recent years [31]. Current cessation systems in Ontario have difficulty reaching youth and young adults and the proportion of youth smokers who tried to quit in the past 12 months has declined since 1999 [31]. Despite that, the youth smoking prevalence still has greater reduction than the adult prevalence indicating the success of youth smoking prevention in Ontario. This fact may also indicate the relative impact of prevention programs in comparison with cessation programs. Since smoking is addictive, cessation is difficult to achieve. Even if the same programs are available to youth and adults, youth may receive more benefit from the fact of not starting cigarette smoking.

Smoking restrictions at home are a leading factor associated with the decline of adult smoking prevalence, but do not appear to be a factor for youth smoking changes. While the analysis does indicate that smoking restrictions at home are associated with more quit smoking attempts, the causal relationship needs to be further tested since it is likely that some social, environmental or policy determinants may result in both the reduction of smoking rates and the increase in home smoking restrictions. For example, quitters may ban smoking in their homes as an aid to staying quit. Nevertheless, since home smoking restrictions are not yet a part of the provincial legislation, the increase of smoking restrictions at home reflects the overall improvement of people’s conception of the harm and social unacceptability of smoking. This conceptual change may be the underlying reason for smoking reduction and stricter rules on smoke-free homes. Further evidence for this explanation is that home smoking restrictions have increased faster than the decrease of smoking prevalence (Table 2), indicating an arrival of conceptual changes before the changes in smoking behaviours.

Although home smoking restrictions do not account for the drop in youth smoking (possibly because youth rarely smoke at home), the analysis shows that the impact of home smoking restrictions on youth smoking has increased (Model 4 in Table 3). This may be another indication of potential success of youth smoking interventions over the years. As discussed earlier, current youth comprehensive tobacco control programs typically focus on reducing the initiation and prevalence of smoking among children and youth. Innovative multi-media campaigns have also been launched to prevent smoking among youth in Ontario [32]. These interventions may all be potential reasons that have led to the drop of youth smoking prevalence and the increased impact of a home smoke-free environment on youth smoking behaviours. Future research is needed to evaluate the impacts of youth smoking policies on local youth smoking behaviours and prevalence.

Geographically, the overall patterns show that northern Ontario residents have higher smoking prevalence for both adults and youth than their southern Ontario counterparts. Since these patterns were obtained after controlling for SES, psycho-social factors, and smoking restrictions, potential reasons for this pattern may be due to a large portion of aboriginal population in northern communities and/or difference in conception of the social and health impacts of smoking between the southern Ontario population and the relatively remote northern communities. Future research may be needed to characterize the conceptual differences between northern and southern Ontario residents or between geographically connected and remote area residents.

While the drop in youth smoking rate was not explained by known factors, such as SES, psycho-social factors and smoking restrictions, the map in Figure 6 actually shows where the highest reductions have been seen. It is suspected that the reduction of smoking prevalence in these areas may be due to the successful implementation of local anti-smoking programs or policies, such as school-based programs [31]. The effective implementation of provincial anti-smoking policies and health promotion strategies actually relies on local Public Health Units to educate, provide appropriate resources to, and communicate with the public through various smoking prevention, protection and cessation programs. These together with other local interventions, mass-media champions, and/or tobacco promotions may lead to the variation in local smoking prevalence.

It can be observed in Figures 4 and 7 that in some places where adult and youth smoking prevalence are high in 2000, the prevalence is even higher in 2008. This suggests that there are areas where existing policies have had no effect. The maps also show that, compared to the adult and youth smoking prevalences in 2000, smoking inequalities among municipalities increased in 2008 although overall smoking rates decreased. Some identified clusters of high smoking prevalence, such as the route staring from Cornwall and along the Ottawa Valley, may indicate potential routes of contraband sales [33]. This phenomenon is somewhat consistent with the temporal models, which also indicate that adult and youth smoking prevalence went up from 2003 to 2008 in homes without smoking restrictions (Figures 1 and 2). These upward trends of smoking prevalence in pro-smoking environments are not explained in the current study, after controlling for demographic, socio-economic, psycho-social and smoking restriction factors. However, excluding the impacts of the above factors, it may be an indication that tobacco sellers’ efforts to promote tobacco products have never stopped, and such efforts may be a potential explanation of these prevalence increases. Further research may be needed to explore the interaction of tobacco sales and pro-smoking environments on smoking behaviours.

Large metropolitan areas, such as the GTA and Ottawa, have the lowest smoking prevalence, while smaller-sized cities have relatively higher smoking prevalence compared to rural areas. Unlike other areas, although the GTA has relatively more smoking reduction, smoking restriction at home is not a leading factor. This may suggest the reduction of smoking rates in GTA may not be due to people’s consciousness about the harm of smoking. The credit is often given to the recent immigrants in GTA since GTA has the largest immigrant population in Canada and recent immigrants have lower smoking rates than non-immigrants [34]. These rural–urban and large-small urban differences need to be addressed in future research.

The study illustrates a more general phenomenon, that the decreased adult and youth smoking prevalence (as shown in Table 2) is actually an averaged result of a dynamic process in which both increasing and decreasing trends exist at different times and in space. The temporal and spatio-temporal analyses used in this research provide an effective method for mapping the variances and interactions between time and place for their impacts on smoking prevalence. The identified spatial and temporal variations help to indicate problems at the local level and suggest future research directions. Identifying these variations helps to strengthen surveillance and monitoring of smoking behaviours and the evaluation of policy and program development at the small-area level. The identified clusters of higher or lower smoking prevalence in particular times and places may help the identification of best practices and area-specific programs for future smoking reduction.
